# Systemic inflammation and extra-articular manifestations in rheumatoid arthritis: a cross-sectional study

**DOI:** 10.3389/fmed.2026.1814238

**Published:** 2026-05-15

**Authors:** Karlygash Sadykova, Gulnaz Nuskabayeva, Ainash Oshibayeva, Shakhnoza Tatykayeva, Elmira Iskandirova, Arzu Mamutova, Aziza Djurabekova, Ugilzhan Tatykayeva

**Affiliations:** 1 Khoja Akhmet Yassawi International Kazakh-Turkish University, Turkistan, Kazakhstan; 2 Samarkand State Medical University, Samarkand, Uzbekistan

**Keywords:** erythrocyte sedimentation rate, extra-articular manifestations, inflammatory markers, real-world study, rheumatoid arthritis, systemic inflammation

## Abstract

**Background:**

Rheumatoid arthritis (RA) is a bodywide inflammatory condition that produces additional symptoms outside the joints that make the disease more severe and lead to patient disability and death. This research investigated various factors associated with the presence of extra-articular manifestations in RA patients.

**Methods:**

The cross-sectional study was conducted from 2023 to 2025 and assessed 299 adult patients who received RA confirmation. Clinical assessments, laboratory tests, and instrumental results were evaluated for extra-articular manifestations, and univariable and multivariable logistic regression analyses were performed to examine these relationships. Results: Extra-articular manifestations were present in 278 patients (93%). Patients with extra-articular manifestations were older (*p* = 0.039) and more frequently had disability (76% vs. 48%, *p* = 0.004). They also demonstrated higher levels of systemic inflammation, including C-reactive protein (median 20 vs. 11 mg/L, *p* = 0.015), erythrocyte sedimentation rate (36 vs. 21 mm/h, *p* < 0.001), rheumatic factor (92 vs. 55.2 IU/mL, *p* < 0.001), and higher antinuclear antibodies index values (1.5 vs. 0.43, *p* = 0.023). In the multivariable model, ESR remained independently associated with extra-articular manifestations (adjusted OR = 1.18, *p* < 0.001), while elevated white blood cell count was inversely associated (adjusted OR = 0.07, *p* = 0.001). Conclusions: Extra-articular manifestations in rheumatoid arthritis are most strongly associated with sustained systemic inflammation, as reflected by ESR, underscoring the importance of monitoring chronic inflammatory burden and the need for longitudinal studies to clarify causality and therapeutic implications.

## Introduction

Rheumatoid arthritis (RA) is a chronic systemic autoimmune disease primarily characterized by inflammatory joint involvement but frequently accompanied by manifestations beyond the musculoskeletal system. These extra-articular manifestations represent an important aspect of RA, as they are associated with increased disease severity, functional disability, and excess morbidity and mortality (1). Extra-articular involvement in RA may affect multiple organ systems, including the cardiovascular, hematological, renal, and immune systems, reflecting the systemic nature of persistent inflammation (2).

Previous studies have explored a range of factors potentially associated with extra-articular manifestations in RA, including demographic characteristics, lifestyle factors, disease severity, inflammatory activity, and immunological markers. Classical markers of inflammation, such as C-reactive protein (CRP) and erythrocyte sedimentation rate (ESR) (1), as well as autoantibodies, including rheumatoid factor (RF) and anti-cyclic citrullinated peptide (anti-CCP) antibodies, have been linked to more severe disease phenotypes and have been investigated as potential correlates of extra-articular involvement (3, 4). However, the strength and consistency of these associations vary across studies, and many associations appear to be influenced by disease duration, treatment exposure, and comorbid conditions.

Importantly, much of the existing evidence is derived from selected cohorts or focuses on specific extra-articular outcomes, limiting the generalizability of findings to routine clinical practice (3, 5). In real-world RA populations, patients often present with substantial heterogeneity in disease characteristics and treatment histories, which may attenuate or modify observed associations between laboratory markers and systemic manifestations. This study aims to examine the associations between demographic, clinical, inflammatory, immunological, and metabolic parameters and the presence of extra-articular manifestations in patients with rheumatoid arthritis.

## Materials and methods

### Study design and population

The cross-sectional analytical study was conducted at the Clinical and Diagnostic Center of Khoja Akhmet Yassawi International Kazak-Turkish University in southern Kazakhstan between 2023 and 2025. Only patients who received a rheumatoid arthritis diagnosis through confirmed medical tests at a university-based clinical and diagnostic center that operated as a routine clinical practice facility were included in the study. Adult participants who were at least 18 years old, had confirmed rheumatoid arthritis, and had complete medical records for evaluation of their joint symptoms were included in the study. Exclusion criteria were the need for immediate medical treatment for their acute condition, those who had severe chronic diseases and psychiatric conditions, and those who were pregnant and had missing information. Of the 460 patients with rheumatoid arthritis, 113 participants declined to participate, and 48 participants had incomplete clinical and laboratory data. Therefore, only 299 patients with RA were included in the analysis.

All patients received treatment according to current clinical guidelines. Therapy included conventional synthetic disease-modifying antirheumatic drugs (DMARDs), primarily methotrexate as first-line treatment, with leflunomide, sulfasalazine, or combination therapy used when indicated. Biological agents were prescribed in selected cases. Patients without extra-articular manifestations generally received standard therapy: DMARDs, non-steroidal anti-inflammatory drugs, and glucocorticoids (6).

### Data collection and covariates

The demographic information about participants’ sex and work status, and their lifestyle details about smoking and drinking, was collected through a standardized questionnaire after participants agreed to share their family medical background and disability information. The clinical examination provided information about joint and extra-articular symptoms, which were evaluated during the assessment.

The certified clinical diagnostic laboratory conducted all laboratory tests, including complete blood counts for hemoglobin, white blood cell count, and platelet count. The laboratory conducted tests for inflammatory markers, including CRP and ESR. The laboratory performed tests to measure RF, anti-CCP antibodies, and antinuclear antibodies (ANA) through an index calculation. ANA testing was performed using ELISA due to routine laboratory availability and should be interpreted only as a supplementary laboratory marker, not as an independent diagnostic criterion.

The laboratory conducted tests to measure serum calcium, glucose, uric acid, total protein, serum iron, and 25-hydroxyvitamin D. Urinalysis was performed, with assessment of proteinuria. The standard clinical evaluation methods of chest X-ray, echocardiography, and electrocardiography served as instrumental examination tools.

### Outcome assessment

The primary outcome was the presence of extra-articular manifestations of rheumatoid arthritis. EAMs were defined as clinically relevant involvement of organs and systems outside the joints, identified through a comprehensive review of inpatient and outpatient medical records, and based on predefined clinical, laboratory, and instrumental criteria. These included: (1) hematological abnormalities such as anemia of chronic disease, leukopenia, thrombocytosis, thrombocytopenia; (2) renal involvement such as proteinuria and/or pathological urinalysis findings; and (3) systemic organ involvement such as cardiovascular, pulmonary, neurological, dermatological, confirmed where possible by instrumental methods, for example, imaging or echocardiography. In this study, extra-articular manifestations were assessed as cumulative, defined as the presence of at least one documented extra-articular manifestation at any time during the disease course prior to or at inclusion.

### Statistical analysis

Continuous variables were analyzed using mean values with standard deviations and median values with interquartile ranges, depending on their distribution. Categorical data were displayed as frequencies and percentages. Between-group comparisons were performed using Student’s t-test or the Mann-Whitney U test for continuous variables and the chi-square test or Fisher’s exact test for categorical variables. Logistic regression was employed to identify which factors are associated with the appearance of extra-articular manifestations. Variables that showed statistical significance in univariable tests entered the multivariable analysis. This study used a two-sided *p*-value analysis to determine statistical significance at the 0.05 significance threshold.

### Ethical approval

The study protocol was approved by the local Ethics Committee of Khoja Akhmet Yassawi International Kazak-Turkish University (Protocol No. 9, November 14, 2022). All participants provided written consent before the study began, in accordance with the Declaration of Helsinki.

## Results

The study included 299 patients, of whom 278 (93%) had extra-articular manifestations, whereas 21 (7%) did not. The patients who developed extra-articular symptoms were older than those without such symptoms (56 ± 13 vs. 50 ± 14 years; *p* = 0.039) (Table 1). The sex distribution between the two groups showed no significant difference, although females accounted for majority of patients without extra-articular manifestations (*p* = 0.082). The two groups maintained identical employment status, drinking habits, and genetic background.

**TABLE 1:** Demographic and clinical characteristics of patients according to the presence of extra-articular manifestations.

Characteristics	Total (*n* = 299)	Extra-articular manifestations		*p*-value
		No (*n* = 21; 7%)	Yes (*n* = 278; 93%)	
Age (y.o.), mean (SD)	56 (13)	50 (14)	56 (13)	0.039
Disease duration (years), mean (SD)	6 (4)	4 (2)	7 (4)	0.007
Gender, n (%)				0.082
Male	46 (15)	6 (29)	40 (14)	
Female	253 (85)	15 (71)	238 (86)	
Work, n (%)				0.247
No	230 (77)	14 (67)	216 (78)	
Yes	69 (23)	7 (33)	62 (22)	
Smoking, n (%)				0.063
No	255 (85)	15 (71)	240 (86)	
Yes	44 (15)	6 (29)	38 (14)	
Alcohol, n (%)				0.595
No	254 (85)	17 (81)	237 (85)	
Yes	45 (15)	4 (19)	41 (15)	
Heredity, n (%)				0.529
No	217 (73)	14 (67)	203 (73)	
Yes	82 (27)	7 (33)	75 (27)	
Disability, n (%)				0.004
No	77 (26)	11 (52)	66 (24)	
Yes	222 (74)	10 (48)	212 (76)	
Chest X-ray, n (%)				0.388 0.413
Normal	258 (86)	17 (82)	241 (87)	
Changes not associated with RA	26 (9)	2 (9)	24 (9)	
Changes associated with RA	15 (5)	2 (9)	13 (4)	
Heart ultrasound, n (%)				
Normal	105 (35)	7 (33)	98 (35)	
Pericarditis	12 (4)	0	12 (4)	
Myocardial dysfunction	95 (32)	10 (48)	85 (31)	
Valvular changes	87 (29)	4 (19)	83 (30)	
Electrocardiogram, n (%)				0.691
Normal	197 (66)	14 (66)	183 (66)	
Minor changes	70 (23)	6 (29)	64 (23)	
Typical changes in RA	32 (11)	1 (5)	31 (11)	

The results of this study show that disability status was significantly associated with the development of extra-articular symptoms because disabled patients accounted for 76% of the extra-articular group, yet disability status occurred in only 48% of patients without extra-articular involvement (*p* = 0.004). No significant differences were observed between groups regarding chest X-ray findings, echocardiographic abnormalities, or electrocardiographic changes.

Among the 299 patients, those with extra-articular manifestations showed a higher systemic inflammatory activity and immune activation than those without extra-articular involvement (Table 2). Patients with extra-articular manifestations had significantly higher CRP levels (median 20 vs. 11 mg/L; *p* = 0.015), RF concentrations (92 vs. 55.2 IU/mL; *p* < 0.001), and ANA index values (1.5 vs. 0.43; *p* = 0.023). ESR was markedly elevated in patients with extra-articular manifestations (36 vs. 21 mm/h; *p* < 0.001). In addition, an elevated white blood cell count was more frequent in this group (19% vs. 38%; *p* = 0.032). In contrast, anti-CCP, as well as Wright-Heddelson reaction positivity, did not differ significantly between groups. Regarding metabolic and biochemical parameters, serum calcium levels were significantly lower in patients with extra-articular manifestations (2.12 vs. 2.22 mmol/L; *p* = 0.016), while uric acid, glucose, total protein, vitamin D, serum iron, and proteinuria showed no significant differences. Hematological parameters, including hemoglobin and platelet count, were comparable between groups.

**TABLE 2:** Inflammatory, metabolic, and hematological parameters according to the presence of extra-articular manifestations.

Parameters	Total (*n* = 299)	Extra-articular manifestations		*p*-value
		No (*n* = 21; 7%)	Yes (*n* = 278; 93%)	
Inflammatory and immunological markers				
CRP, mg/L	20 (11.1–30)	11 (7.37–19.6)	20 (11.7–30.4)	0.015
Anti-CCP, U/mL	133.5 (10.3–165)	142 (10.3–170)	132 (10.3–165)	0.696
RF, IU/mL	92 (52–115)	55.2 (12.5–78.6)	92 (56–118)	< 0.001
ANA, index	1.5 (0.4–5.4)	0.43 (0.37–1.7)	1.5 (0.41–5.6)	0.023
Wright–Heddelson reaction, n (%)				0.430
Negative	271 (91)	18 (86)	253 (91)	
Positive	28 (9)	3 (14)	25 (9)	
Metabolic and biochemical parameters				
Uric acid, μmol/L	298 (275–319)	310 (280–373)	298 (275–315)	0.370
Fasting plasma glucose, mmol/L	5.33 (5.1–7.8)	5.4 (5.02–6.19)	5.3 (5.1–7.8)	0.739
Total protein, g/L	72.6 (69.7–87.2)	75.5 (70–87.4)	72.5 (69.7–87)	0.700
25(OH)D, ng/mL	24.8 (18–29)	24 (15.8–29)	25 (18.2–29)	0.413
Serum calcium, mmol/L	2.12 (1.9–2.22)	2.22 (2.12–2.29)	2.12 (1.9–2.22)	0.016
Serum iron, μmol/L	9.5 (4.8–10.8)	9.6 (7.8–10.8)	9.4 (4.6–10.8)	0.782
Proteinuria, n (%)				0.605
Absent	267 (89)	19 (90)	248 (89)	
Present	32 (11)	2 (10)	30 (11)	
Hematological parameters				
ESR, mm/h	36 (28–40)	21 (15–26)	36 (29–40)	< 0.001
Hb, g/L	113 (109–118)	113 (109–119)	114 (109–118)	0.928
PLT, × 10^9^/L	395 (336–410)	392 (305–404)	397 (348–410)	0.181
WBC, n (%)				0.032
Normal	239 (80)	13 (62)	226 (81)	
Elevated	60 (20)	8 (38)	52 (19)	

CRP, C-reactive protein; anti-CCP, Anti–cyclic citrullinated peptide antibodies; RF, Rheumatoid factor; ANA, Antinuclear antibodies; 25(OH)D, 25-hydroxyvitamin D; ESR, Erythrocyte sedimentation rate; Hb, Hemoglobin; PLT, Platelet count; WBC, White blood cell count.

Unadjusted and multivariable logistic regression analyses were performed to identify factors associated with the presence of extra-articular manifestations. Only variables demonstrating statistical significance in univariable analyses were included in the model (Table 3), while non-significant variables are presented in Supplementary Table 1. In the unadjusted analysis, increasing age was associated with higher odds of extra-articular manifestations (OR = 1.03, *p* = 0.042). Each additional year of disease duration increased the odds of extra-articular manifestations by 26% (*p* = 0.009). Disability status was strongly associated with extra-articular involvement (OR = 3.53, *p* = 0.006). Higher levels of systemic inflammatory markers, including CRP, RF, and ESR, were also significantly associated with extra-articular manifestations.

**TABLE 3:** Logistic regression analysis of factors associated with extra-articular manifestations.

Predictors	Unadjusted model		Adjusted model	
	OR (95% CI)	*p*-value	OR (95% CI)	*p*-value
Age	1.03 (1.01–1.07)	0.042	0.70 (0.36–1.35)	0.286
Disease duration	1.26 (1.06–1.49)	0.009	1.71 (1.24–2.35)	0.001
Disability [ref. no]				
Yes	3.53 (1.44–8.69)	0.006	4.14 (0.51–9.36)	0.131
CRP	1.05 (1.01–1.08)	0.028	1.05 (1.01–1.10)	0.025
RF	1.02 (1.01–1.04)	< 0.001	1.01 (0.99–1.02)	0.062
Serum calcium	0.11 (0.01–0.80)	0.030	0.60 (0.03–12.3)	0.743
ESR	1.13 (1.08–1.19)	< 0.001	1.18 (1.09–1.27)	< 0.001
WBC (ref. normal)				
Elevated	0.37 (0.15–0.95)	0.038	0.07 (0.02–032)	0.001

After multivariable adjustment, most associations observed in the unadjusted model were attenuated. Age, disability status, RF, and serum calcium were no longer independently associated with extra-articular manifestations. In contrast, disease duration remained an independent predictor of extra-articular manifestations after adjustment (adjusted OR = 1.71, *p* = 0.001). ESR remained a strong independent predictor (adjusted OR = 1.18, *p* < 0.001), indicating a robust association between sustained systemic inflammation and extra-articular involvement. Additionally, elevated WBC count remained independently associated with lower odds of extra-articular manifestations (adjusted OR = 0.07, *p* = 0.001).

## Discussion

The research investigated demographic variables, clinical data, inflammatory markers, and immunological factors that affect extra-articular manifestations in rheumatoid arthritis patients using a single-time data collection approach. The main discovery is that ESR and WBC count were the only two factors that maintained independent associations with extra-articular involvement after multivariable analysis. The research indicates that systemic inflammation markers show stronger associations with RA extra-articular symptoms than any other factor, including demographic traits and autoantibody presence, in patients with RA in the general population. Patients with extra-articular manifestations had a longer disease duration compared to those without extra-articular involvement.

### Systemic inflammation and extra-articular manifestations

The current practice of using ESR alone to predict extra-articular manifestations in RA patients shows that chronic inflammation serves as the main factor that causes RA to develop systemic symptoms (7–9). The ESR inflammatory activity shows patterns different from those of CRP because it is less affected by short-term changes and acute medication responses (7). Research studies have shown that high ESR values indicate severe RA complications, which lead to extra-articular symptoms and elevated mortality risk (8, 9). Our findings support this concept and suggest that ESR may better capture the cumulative inflammatory exposure relevant to the development of extra-articular manifestations than more dynamic markers such as CRP.

CRP levels were higher in patients with extra-articular manifestations, as shown by both univariate and multivariate tests. The decrease in CRP levels might result from patients who continue receiving anti-inflammatory or immunosuppressive treatment, which lowers their CRP values even though their bodies continue to show signs of systemic disease (10, 11). Research studies using real population groups demonstrated that CRP levels correlated with extra-articular symptoms only after controlling for potential confounding variables (11). Importantly, treatment with DMARDs, glucocorticoids, and other agents may substantially influence inflammatory markers and laboratory parameters (12), potentially attenuating associations observed in multivariable models.

### White blood cell count and immune response

The adjusted model showed an unexpected yet steady association between WBC levels and the development of extra-articular symptoms, with these symptoms becoming less common as WBC levels increased. Elevated WBC count on blood tests usually indicates inflammation, but it can also result from acute inflammatory reactions and infections, as well as from medical treatments that include glucocorticoid administration (13). The development of extra-articular manifestations in RA results from long-term immune dysfunction rather than short-term inflammatory reactions (14). The inverse association observed in this study may therefore indicate that patients with established extra-articular involvement represent a subgroup with more chronic, smoldering inflammation rather than acute leukocytosis. Given the unexpected inverse association observed in the adjusted model, this finding should be interpreted cautiously. Possible explanations include treatment effects, particularly glucocorticoid-induced leukocytosis (15, 16), differences between acute and chronic inflammatory responses, and residual confounding (17). The research results could stem from remaining confounding factors or treatment side effects, which need careful evaluation (12, 18). In current study, given the substantial imbalance between groups and the small size of the non-EAM group, the observed effect estimate may be unstable and potentially exaggerated. Therefore, this association should be interpreted with caution and considered hypothesis-generating, requiring confirmation in larger and more balanced cohorts.

### Autoantibodies and immunological markers

The univariable analyses showed that patients with extra-articular manifestations had significantly elevated rheumatoid factor and ANA index values, as previously reported to indicate more severe and widespread disease (3, 19). The research found that autoantibody status served as a disease severity indicator rather than an independent factor that causes extra-articular symptoms (3). Anti-CCP antibodies, which are strongly associated with erosive joint disease, were not associated with extra-articular manifestations in this study. Research indicates that anti-CCP antibodies show a stronger association with joint damage progression than with extra-articular diseases, which affect the body beyond joints (18, 20).

### Metabolic parameters and mineral metabolism

Patients with extra-articular manifestations exhibited lower serum calcium levels in univariable analyses; however, this finding should be interpreted cautiously and considered exploratory and hypothesis-generating, requiring confirmation in future studies. The chronic inflammatory state in RA patients leads to disturbances in calcium homeostasis and bone metabolism, as their bodies produce cytokines that interfere with these systems (21, 22). The multivariable model showed that serum calcium failed to maintain its independent assocition with extra-articular manifestations, suggesting that the association may stem from general inflammatory processes rather than reflecting an independent factor.

The metabolic parameters of glucose, uric acid, vitamin D, and serum iron did not show any significant relationship with extra-articular manifestations. The research indicates that metabolic problems do not significantly affect the development of extra-articular symptoms when compared to inflammatory conditions in this study population (3).

### Clinical and demographic factors

The research established an association between disability status and additional joint problems because patients with systemic involvement develop more severe disease, which results in worsening functional abilities (3). The relationship between disability and chronic inflammation became weaker after researchers made their adjustments, which showed that disability develops from chronic inflammation instead of being an independent risk factor (3, 23). The univariate analysis showed that age was associated with extra-articular manifestations, but this association disappeared after model adjustment, suggesting that age-related effects lose significance when researchers include inflammatory burden and disease severity in their analysis (8).

The study found no meaningful relationships between any of the following variables: sex, employment status, smoking, alcohol use, and instrumental test results from chest X-ray, echocardiography, and electrocardiography. The study results confirmed earlier real-world studies showing that demographic and lifestyle factors do not affect extra-articular manifestations, as researchers analyzed inflammatory disease activity (1, 24).

### Strengths and limitations

The research benefits from its investigation of a specific RA patient group, its comprehensive evaluation of inflammatory markers, immunological, and metabolic indicators, and its use of multivariable models to manage confounding factors. The research findings stem from typical medical procedures, which makes them applicable to actual healthcare environments.

The research contains multiple restrictions that need to be recognized. The study design prevents researchers from establishing cause-and-effect relationships because it does not enable them to track how inflammation develops into extra-articular manifestations over time. The relatively small number of patients without extra-articular involvement may have limited statistical power for some comparisons. In addition, the substantial imbalance between groups may have affected the stability of multivariable estimates and increased the risk of overfitting, particularly in the context of a relatively small comparison group. Furthermore, the cumulative definition of extra-articular manifestations, without detailed information on disease duration, disease activity, and timing of manifestation onset, limits the ability to account for disease trajectory and may influence the observed associations. It should be noted that the relatively high prevalence of EAMs observed in this study may be partially explained by the inclusion of laboratory abnormalities, especially hematological changes, which are not consistently classified as extra-articular manifestations in all studies. The study failed to consider treatment-related factors, including medication types and their durations, because these factors could have affected the laboratory test results. The study defined extra-articular manifestations by analyzing clinical records, laboratory results, and instrumental tests, which might not capture all possible systemic symptoms. Finally, these findings should be interpreted cautiously and considered exploratory.

## Conclusion

In this real-world study of patients with rheumatoid arthritis, extra-articular manifestations were most strongly associated with markers of sustained systemic inflammation. After multivariable adjustment, disease duration, CRP, ESR and WBC remained as the independent predictors of extra-articular involvement, while associations with demographic factors, autoantibodies, and metabolic parameters were attenuated. These findings highlight the central role of chronic inflammatory burden in extra-articular disease in rheumatoid arthritis and emphasize the need for focused inflammatory monitoring in clinical practice.

## Data Availability

The original contributions presented in the study are included in the article/Supplementary material, further inquiries can be directed to the corresponding author.

## References

[1] Figus FA Piga M Azzolin I McConnell R Iagnocco A. Rheumatoid arthritis: extra-articular manifestations and comorbidities. *Autoimmun Rev*. (2021) 20:102776. 10.1016/j.autrev.2021.102776 33609792

[2] Marcucci E Bartoloni E Alunno A Leone MC Cafaro G Luccioli F et al. Extra-articular rheumatoid arthritis. *Reumatismo.* (2018) 70:212–24. 10.4081/reumatismo.2018.1106 30570239

[3] Bonfiglioli KR de Medeiros Ribeiro AC Carnieletto AP Pereira I Domiciano DS da Silva HC et al. Extra-articular manifestations of rheumatoid arthritis remain a major challenge: data from a large, multi-centric cohort. *Adv Rheumatol.* (2023) 63:34. 10.1186/s42358-023-00318-y 37496102

[4] Conforti A Di Cola I Pavlych V Ruscitti P Berardicurti O Ursini F et al. Beyond the joints, the extra-articular manifestations in rheumatoid arthritis. *Autoimmun Rev*. (2021) 20:102735. 10.1016/j.autrev.2020.102735 33346115

[5] Kimbrough BA Crowson CS Davis JM Matteson EL Myasoedova E. Decline in incidence of extra-articular manifestations of rheumatoid arthritis: a population-based cohort study. *Arthritis Care Res*. (2024) 76:454–62. 10.1002/acr.25231 PMC10924769 37691141

[6] Turdalin N Gabdulina G Aubakirova B Amanzholova A Smagulova G. *Rheumatoid Arthritis. Version: Clinical Protocols of the Ministry of Health of the Republic of Kazakhstan - 2016 [Internet].* (2016). Available online at: https://diseases.medelement.com/disease/%D1%80%D0%B5%D0%B2%D0%BC%D0%B0%D1%82%D0%BE%D0%B8%D0%B4%D0%BD%D1%8B%D0%B9-%D0%B0%D1%80%D1%82%D1%80%D0%B8%D1%82-2016/14947 (accessed March 18, 2026).

[7] Chen X Zhang M Wang T Li Y Wei M. Influence factors of extra-articular manifestations in rheumatoid arthritis. *Open Med*. (2020) 15:787–95. 10.1515/med-2020-0217 33313414 PMC7706136

[8] Chirila RM Berianu F Abril A Butendieck RR. Extra-articular involvement of rheumatoid arthritis in three seropositive patients in the absence of initial joint involvement. *Immun Inflamm Dis.* (2021) 9:1613–7. 10.1002/iid3.514 34473908 PMC8589363

[9] Ljung L Jönsson E Franklin J Berglin E Lundquist A Rantapää-Dahlqvist S. Incidence and predisposing factors of extra-articular manifestations in contemporary rheumatoid arthritis. *Eur J Intern Med*. (2024) 126:95–101. 10.1016/j.ejim.2024.04.026 38705755

[10] Pope JE Choy EH. C-reactive protein and implications in rheumatoid arthritis and associated comorbidities. *Semin Arthritis Rheum.* (2021) 51:219–29. 10.1016/j.semarthrit.2020.11.005 33385862

[11] Rudi T Zietemann V Meissner Y Zink A Krause A Lorenz HM et al. Impact of DMARD treatment and systemic inflammation on all-cause mortality in patients with rheumatoid arthritis and interstitial lung disease: a cohort study from the German RABBIT register. *RMD Open.* (2024) 10:e003789. 10.1136/rmdopen-2023-003789 38580343 PMC11002391

[12] Kharbanda H Khanna M Thakrar K Basak M Dwivedi M Das PP. Effect of DMARD therapy on inflammatory biomarkers and disease activity in rheumatoid arthritis patients. *J Orthop Case Rep*. (2025) 15:299–305. 10.13107/jocr.2025.v15.i04.5526 PMC11981487 40212483

[13] Barbulescu A Sjölander A Delcoigne B Askling J Frisell T. Glucocorticoid exposure and the risk of serious infections in rheumatoid arthritis: a marginal structural model application. *Rheumatology.* (2023) 62:3391–9. 10.1093/rheumatology/kead083 36821426 PMC10547528

[14] Mitroviæ J Hrkaè S Teèer J Golob M Ljilja Posavec A Kolar Mitroviæ H et al. Pathogenesis of extraarticular manifestations in rheumatoid arthritis-a comprehensive review. *Biomedicines*. (2023) 11:1262. 10.3390/biomedicines11051262 37238933 PMC10216027

[15] Petrackova A Horak P Savara J Skacelova M Kriegova E. High cumulative glucocorticoid dose is associated with increased levels of inflammation-related mediators in active rheumatoid arthritis. *Front Immunol.* (2024) 15:1505615. 10.3389/fimmu.2024.1505615 39744635 PMC11688219

[16] Syed KM Pinals RS. Leukocytosis in rheumatoid arthritis. *J Clin Rheumatol*. (1996) 2:197–202. 10.1097/00124743-199608000-00007 19078065

[17] Al-Ghamdi A Attar SM. Extra-articular manifestations of rheumatoid arthritis: a hospital-based study. *Ann Saudi Med.* (2009) 29:189–93. 10.4103/0256-4947.51774 19448378 PMC2813651

[18] Harrold LR Bryson J Lehman T Zhuo J Gao S Han X et al. Association between baseline anti-cyclic citrullinated peptide antibodies and 6-month clinical response following abatacept or TNF inhibitor treatment: a real-world analysis of biologic-experienced patients with RA. *Rheumatol Ther.* (2021) 8:937–53. 10.1007/s40744-021-00310-2 34047953 PMC8217398

[19] Muhammad S Khan AM Amin R Khan A Yaseen M Muhammad H. The association of anti-cyclic citrullinated peptide positivity with extra-articular manifestations in patients with rheumatoid arthritis. *IJBR.* (2025) 3:630–3. 10.70749/ijbr.v3i7.2015

[20] Maeda K Yoshida K Nishizawa T Otani K Yamashita Y Okabe H et al. Inflammation and bone metabolism in rheumatoid arthritis: molecular mechanisms of joint destruction and pharmacological treatments. *Int J Mol Sci.* (2022) 23:2871. 10.3390/ijms23052871 35270012 PMC8911191

[21] Liang HY Yin HX Li SF Chen Y Zhao YJ Hu W et al. Calcium-permeable channels cooperation for rheumatoid arthritis: therapeutic opportunities. *Biomolecules*. (2022) 12:1383. 10.3390/biom12101383 36291594 PMC9599458

[22] Clasen JL Cole R Aune D Sellon E Heath AK. Vitamin D status and risk of rheumatoid arthritis: systematic review and meta-analysis. *BMC Rheumatol.* (2023) 7:3. 10.1186/s41927-023-00325-y 36918989 PMC10015722

[23] Lee KA Lee B Kim HS. Risk of ILD and safety outcomes across DMARD classes in rheumatoid arthritis and RA-ILD. *Semin Arthritis Rheum*. (2026) 77:152921. 10.1016/j.semarthrit.2026.152921 41534248

[24] Mikuls TR Baker JF Cannon GW England BR Kerr G Reimold A. The veterans affairs rheumatoid arthritis registry: a unique population in rheumatoid arthritis research. *Semin Arthritis Rheum.* (2025) 70S:152580. 10.1016/j.semarthrit.2024.152580 39580339 PMC12937311

