# Supplementary material for: Systemic inflammation and extra-articular manifestations in rheumatoid arthritis: a cross-sectional study
Source: Front Med (Lausanne). 2026 May 15;13:1814238. doi: 10.3389/fmed.2026.1814238 (PMC13218873; doi:10.3389/fmed.2026.1814238)
Supplement: Supplementary file 1 [file Table_1.docx]

**Supplementary table 1. Non-significant factors were excluded from the Logistic regression.**

|  | **Unadjusted model** | |
| --- | --- | --- |
|  | **OR [95% CI]** | **p-value** |
| Gender [ref. male] |  |  |
| Female | 2.38 [0.87-6.49] | 0.091 |
| Work [ref. no] |  |  |
| Yes | 0.57 [0.22-1.48] | 0.252 |
| Smoking [ref. no] |  |  |
| Yes | 2.53 [0.92-6.91] | 0.071 |
| Alcohol [ref. no] |  |  |
| Yes | 0.74 [0.25-2.30] | 0.596 |
| Heredity [ref. no] |  |  |
| Yes | 0.74 [0.29-1.90] | 0.530 |
| Chest X-ray [ref. normal] |  |  |
| Changes not associated with RA | 0.85 [0.18-3.89] | 0.830 |
| Changes associated with RA | 0.46 [0.09-2.19] | 0.330 |
| Electrocardiogram [ref. normal] |  |  |
| Minor changes | 0.82 [0.30-2.21] | 0.690 |
| Typical changes in RA | 2.37 [0.30-18.7] | 0.412 |
| Anti-CCP | 0.99 [0.99-1.00] | 0.541 |
| ANA | 1.16 [0.98-1.37] | 0.091 |
| Wright–Heddelson reaction [ref. negative] |  |  |
| Positive | 0.59 [0.16-2.15] | 0.427 |
| Uric acid | 0.99 [0.99-1.00] | 0.524 |
| Fasting plasma glucose | 1.08 [0.89-1.29] | 0.427 |
| Total protein | 0.99 [0.95-1.04] | 0.786 |
| 25(OH)D | 1.04 [0.98-1.10] | 0.216 |
| Serum iron | 0.96 [0.89-1.03] | 0.280 |
| Proteinuria [ref. absent] |  |  |
| Present | 1.15 [0.26-5.18] | 0.856 |
| Hb | 0.99 [0.96-1.02] | 0.474 |
| PLT | 1.00 [0.99-1.01] | 0.151 |
